# Community and individual level determinants and spatial distribution of deworming among preschool age children in Ethiopia: spatial and multi-level analysis

**DOI:** 10.1186/s12889-022-13249-y

**Published:** 2022-05-02

**Authors:** Daniel Gashaneh Belay, Melaku Hunie Asratie, Moges Gashaw, Nuhamin Tesfa Tsega, Mastewal Endalew, Fantu Mamo Aragaw

**Affiliations:** 1 Department of Human Anatomy, School of Medicine, College of Medicine and Health Sciences, University of Gondar, Gondar, Ethiopia; 2 Department of Epidemiology and Biostatistics, Institute of Public Health Health, College of Medicine and Health Sciences, University of Gondar, Gondar, Ethiopia; 3 Department of Women’s and Family, School of Midwifery, College of Medicine and Health Sciences, University of Gondar, Gondar, Ethiopia; 4 Department of physiotherapy, College of Medicine and Health Sciences, University of Gondar, Gondar, Ethiopia; 5 Department of Environmental and Occupational Health and Safety, Institute of Public Health, College of Medicine and Health Science, University of Gondar, Gondar, Ethiopia

**Keywords:** Deworming, Preschool, Spatial, Ethiopia

## Abstract

**Background:**

Soil-transmitted helminths caused millions of morbidity of preschool age children in sub-Saharan Africa with low socio-economic status and lack of clean water and sanitation. In Ethiopia, nearly half of children are affected by intestinal parasites. Despite this prevalence, deworming medication utilization among preschool age children is low. Hence, this study aimed to assess the community and individual level determinants and spatial distributions of deworming among preschool age children in Ethiopia.

**Methods:**

Crossectional collected 2016 Ethiopian Demographic and Health Survey datasets with a total weighted 8146 children 12–59 months old were used for this study. The data were cleaned, extracted, and analyzed using STAT Version 16 software and exported to MS excel for spatial analysis. In addition, ArcGIS and SaTScan software were used to detect the geographic distribution of deworming utilization among preschool age children.

**Results:**

The magnitude of deworming among preschool age children in Ethiopia was 13.32% (95% CI: 12.60, 14.08) and ranges from the lowest 3.34% (95% CI: 1.01, 10.45) Afar region to the highest 28.66% (95% CI:24.95, 32.69) Tigray region. In multilevel multivariable logistics regression analysis; variables such as secondary and above women education [AOR = 1.89; 95%CI; 1.32, 2.73], women who have occupation [AOR = 1.47; 95%CI; 1.23, 1.76], child with 12–23 months old [AOR = 2.00; 95%CI; 1.62, 2.46], having ANC visit [AOR = 1.68; 95%CI; 1.35, 2.08], households that have media exposure [AOR = 1.50; 95%CI; 1.22, 1.85] were significantly associated with deworming among preschool age children. Afar, Eastern Amhara, Dire Dewa, Harari, Somalia, and Eastern SNNPE regions were cold spot regions with Global Moran’s I value 0.268 (*p* < 0.0001) for deworming of preschool age children.

**Conclusions:**

The prevalence of deworming among preschool age children in Ethiopia is relatively low. Individual-level factors such as; maternal education and occupation, having ANC visit, child age, household media exposure, and community-level variables such as; community media usage had a significant association with deworming among preschool age children in Ethiopia. These findings highlight that, the Ministry of Health (MOH) Ethiopia should prepare a regular campaign for deworming programs for preschool age children. Mass media promotion of deworming should be strengthened. The Ministry of Education should work to strengthen women’s education, household and community media exposure. Prior attention should be given to low deworming regions such as Afar, Somalia, Diredewa, and Harari regions.

## Background

Playing in the sand and getting dirty is a part of growing up, but millions of children in underdeveloped nations are in danger of obtaining soil-transmitted helminths (STH) as a result of these childhood fun activities [1]. There are four main soil-transmitted helminths which are hookworm (Ancylostoma duodenal and Necatoramericanus), roundworm (Ascaris lumbricoides), and whipworm (Trichuris trichiura) [2, 3].

It is estimated that STH affects more than 2 billion people worldwide, and, 90% of whom are living in sub-Saharan Africa [4] 10–15% of whom are children of preschool age [5, 6]. A systematic review and meta-analysis conducted in Ethiopia showed that the pooled prevalence of intestinal parasites among pre-school and school children was 48% [7], and worsen the Amhara region (65.6%) [8].

The disease affects the poorest of the poor particularly abundant among people living in rural or deprived urban settings with low socio-economic status, lack of clean water sanitation [3, 4]. Compared with any other age group, school-aged children and preschool children are the most vulnerable group and they harbor the greatest numbers of intestinal worms. This is because of the daily rituals they play in fecal contaminated soil and their weak immunity and needs special care and follow-up [4, 9]. Therefore, this diversified illness caused millions of morbidity among under-five children who live in developing countries [10]. They also experience growth stunting, anemia [11], and diminished physical fitness as well as impaired memory and cognition [4, 9]. The economic and social consequences of helminthic infections go far beyond the obvious health impacts, including lost school attendance and productive working time [6].

The World Health Organization (WHO) recommends treating all preschool age children as of 12 months of old, at regular intervals with deworming drugs in areas where helminth infection is common [3, 12]. This strategic plan was eliminating STH as a public health problem in children by 2020 [13] focusing on mass treatment with broad-spectrum anthelminthic drugs [4, 14].

Global deworming programs aim to reach 75% of at-risk preschool-age children (pre-SAC) by 2020 [5, 13]. But the mean global deworming coverage in pre-school children in 50 soil-transmitted helminths (STH)-endemic countries was estimated at 36% between 2004 and 2017 [15] and progressively declined in four consecutive years 37.1% in 2010, 30.6% in 2011, 24.7% in 2012 and 23.9% in 2013 [2]. On the other hand, data collected from 39 countries’ UNICEF offices showed that deworming coverage among pre-SAC increased to 49.1% [5]. Whereas in Ethiopia, the prevalence of deworming among 24–59 months old children was 15.1% [16].

Many countries, including Ethiopia, have been launching selected deworming programs to control intestinal geo-helminthic infections among preschool-age children to reduce their morbidity and mortality [4]. But its implementation was low [16]. Studies showed that factors such as; maternal media exposure status [1, 12, 16, 17], maternal control of household healthcare decisions [16], child vitamin-A supplementation [16], having a history of diarrheal disease [16], maternal and paternal education [16, 18, 19], and child age [12] were significant predictors of deworming supplements.

In Ethiopia, even if deworming supplementation in children takes place at the community level based on campaign, previous studies on utilization of deworming medication among children were done using individual level factor analysis only [16]. This assumes that there is no community effect beyond the characteristics of individuals [20, 21]. Therefore, the impact of community-level factors on deworming among preschool age children (pre SAC) remains understudied [16]. Moreover, analyzing the hierarchical nature data like the DHS data using single-level analysis leads to incorrect estimation of parameters and standard errors [22]. Therefore doing multi-level analyses using cluster effect can fill this gap. On the other side, there is scarce evidence in the spatial distribution of deworming supplementation among children in the country.

Therefore this study aimed to assess the magnitude, the individual level, and community level factors and the spatial distribution of deworming utilization among pre SAC in Ethiopia.

## Methods

### Study design, setting, and period

We used cross-sectional data from Ethiopia Demographic and Health Survey (EDHS 2016) for this study. Ethiopia is a sub-Saharan African country with 1.1 million Sq. km coverage and the second-most populous country in Africa with an estimated population of 114,963,588 in 2021 [23]. Administratively, Ethiopia is federally decentralized into two city administrations and nine regions [23]. The datasets are publicly available from the DHS website www.dhsprogram.com [24]. The surveys are nationally representative of the country and population-based with large sample sizes [25].

### Populations

The source population was all preschool age children (aged 12–59 months) preceding five years of the survey period in Ethiopia whereas, the study population was preschool age children preceding five years of the survey period in the selected primary sampling unit (PSU). Mothers who had more than one child within the two years preceding the survey were asked questions about the most recent child [25].

Based on DHS recode manual, recent birth children who were died were excluded from the study. However, missing values and “don’t know” responses on whether the child took drugs for intestinal parasites in the last six months preceding the interview are included in the study but considered as not dewormed [25].

Weighted values were used to restore the representativeness of the sample data and calculated from children’s records or kid’s records (KR) EDHS 2016 datasets. Finally, a total weighted sample of 8146 children in the age category of 12–59 months was included in this study.

### Sampling method

Using the 2007 Population and Housing Census (PHC) as a sampling frame, the EDHS used a stratified two-stage cluster sampling technique. Stratification was achieved by separating every eleven regions of Ethiopia into rural and urban areas. In total, 21 sampling strata have been created (except the Addis Abeba region which is only urban). Therefore, in the first stage, 645 Enumeration Areas (EAs) (202 in the urban area and 443 in the rural) were selected with probability selection proportional to the size of EA. In selected EAs, households (HHs) comprise the second stage of sampling. In the second stage, after listing the households, on average, 28 households have been selected using equal probability systematic sampling in the selected EAs [26]. The detailed sampling procedure was available in each DHS report from the Measure DHS website [24].

### Study variables

The outcome variable of this study was taking deworming medication by preschool aged children. During the survey, their mother was asked questions about their under five years children who take drugs for intestinal parasites in the last six months preceding the interview [25].

Individual and community-level independent variables have been studied. The individual-level factors include socio-demographic characteristics such as; the age of the mother, mother employment, marital status, family size, maternal education, media exposure, and household wealth status were included. Child-related factors such as the age of the child, sex of the child, the plurality of birth, and birth order are all taken into account. Health service utilization-related factors such as place of delivery, pregnancy wontedness, and ANC visit were also considered. The community-level factors include; distance from health facilities, community media exposure, community poverty level, community women education, place of residence, and region were considered.

Media exposure was created from three variables; listening to the radio, watching TV, and reading newspapers. If a woman has at least one type of media exposure, she was considered exposed to media [27]. Whereas, community-level media exposure was assessed using the proportion of women who had at least been exposed to one media; television, radio, or newspaper. It was coded as “0” for low (communities in which < 50% women had media exposure at least for one media), “1” for high community-level media exposure (communities in which ≥50% women had at least for one media [28, 29]. Community level poverty was also determined using the proportion of women in the poorer and poorest quintiles obtained from the wealth index results. It was coded as “0” for low (communities in which < 50% women had poor and poorest wealth quintiles), “1” for high (communities in which ≥50% women had poorest and poorer wealth quintiles) poverty communities [28, 29]. Community-level women’s education was also assessed by the proportion of women who had at least primary education. It was coded as “0” for low (communities in which < 50% women had at least primary education), “1” for high community-level women education (communities in which ≥50% women had at least primary education (at cluster level) [28, 29].

Based on the development status and the need for governmental support, the 11 regions of Ethiopia are categorized into three groups; large central (Tigray, Amhara, Oromia, SNNPR), “small peripherals” (Afar, Benishangul Gumuz, Gambelia and Somali), and ‘three Metropolis’ (Addis Ababa, Harari, and Diredewa) [27].

### Data collection tools and quality control

Demographic and Health Survey (DHS) surveys collect data through different types of questionaries using interviewer administer questionnaire techniques. The missing values in the outcome variables were clearly defined by the DHS guideline [25]. But variables that have a missing value greater than 5% in explanatory variables were dropped from further analysis since complete case analysis is a better missing data management in a crossectional study. The data extractions were performed by public health experts who have experience with DHS data to ensure the quality.

### Data processing and analysis

This study was performed based on the DHS data obtained from the official DHS measure website www.measuredhs.comafter permission has been obtained via an online request by specifying the objectives. The standard DHS dataset was downloaded in STATA format then cleaned, integrate, transformed, and append to produce favorable variables for the analysis. Microsoft Excel and STATA 16 software were used to generate both descriptive and analytic statistics to describe variables in the study using statistical measurements.

### Model building for multi-level analysis

Since the DHS data has hierarchical nature, children were nested within a cluster which violates the standard logistic regression model assumptions such as the independence and equal variance assumptions, a multilevel binary logistic regression model was fitted. Four models were fitted for multi-level analysis. The first was the null model (Model 1) which contained only the outcome variables. It is used to check the variability of deworming utilization across the cluster. The second (Model 2) and the third (Model 3) multilevel models contain individual-level variables and community-level variables respectively. In the fourth model (Model 4), both individual and community level variables were fitted simultaneously with the prevalence of deworming utilization. Model comparisons were done with the standard logistics regression model using the Log-likelihood and deviance test and the model with the highest log-likelihood and lowest deviance was selected as the best-fitted model. The variance inflation factor (VIF) was used to detect multicollinearity, and a variable that has a VIF result of 10 and above is regarded as indicating having multicollinearity [30]. But in this study, all variables had VIF values less than five and the mean VIF value of the final model was 1.50. In the fixed effect measure of association, the variable which has significant association in Adjusted Odds Ratio (AOR) ratios was declared using a *p*-value of < 0.05 with 95% confidence intervals. The random effect used to measure the variation was estimated using the median odds ratio (MOR), Intra Class Correlation Coefficient (ICC), and Proportional Change in Variance (PCV) [29, 31, 32].

### Spatial analysis

Global Moran’s I statistic spatial autocorrelation measure was used to assess the spatial distribution of deworming among preschool age children in Ethiopia [33]. Getis-Ord Gi* statistic hot spot analysis was used to show significant cold spot area for deworming among 12–59 months of children. The proportion of children taking deworming medication among 12–59 month old children in each cluster was taken as an input for cold spot analysis. To predict deworming utilization among preschool children in Ethiopia for unsampled areas based on sampled clusters, the Inverse Distance Weighted (IDW) type spatial interpolation technique was used. Bernoulli based model spatial scan statistics were employed to determine the geographical locations of statistically significant clusters for not dewormed preschool aged children using Kuldorff’s SaTScan version 9.6 software [34]. The scanning window that moves across the study area in which children who had not taken deworming medication were taken as cases and those children who had taken deworming medication were taken as controls to fit the Bernoulli model.

## Results

### Socio demographic characteristics of mothers or caregivers

A total weighted sample of 8146 children of age 12–59 months were included in this study. More than half (54.19%) of mothers of children were found in the age group of 20–34 years, with a median age of 29 (IQR: 25, 35) years. More than three-fifths of women (67.96%) had no formal education. Three-fourths (75.4%) of the children were older than two years. Most of the respondents were live in rural (89.23%) and large central regions (90.22%) [Table 1].

**Table 1:** Socio-demographic characteristics of the mothers/caregivers and the children in a study of trend and determinants of deworming among 6–59 months children in Ethiopia: based on 2016 EDHS

Variables	Categories	Weighted Frequency (n)	Weighted Percentage (%)
Socio-demographic characteristics and health service utilization of the mothers			
Age of women (years)	15–19	1568	19.24
	20–34	4415	54.19
	35–49	2164	26.56
Sex of household head	Male	7926	85.99
	Female	1291	14.01
Educational attainment of women	No education	5536	67.96
	Primary education	2069	25.4
	Secondary & above	541	6.64
Occupation of women	Not working	4377	53.73
	Worked	3769	46.27
Marital status of a mother	Married	7636	93.73
	Not married	511	6.27
Household family size	1–4	2039	25.03
	5–10	5842	71.72
	> 11	265	3.25
Media exposure	No	5519	67.75
	Yes	2627	32.55
Wealth index	Poorest	3858	47.36
	Middle	1702	20.89
	Richest	2587	31.75
Pregnancy wontedness	Wanted	5804	71.24
	Unwanted	2343	28.76
ANC visits	No ANC	1946	38.61
	At least one ANC	3094	61.39
Place of delivery	Home delivery	6262	76.86
	Health facilities	1885	23.14
Child related characteristics			
Sex of child	Male	4241	52.06
	Female	3905	47.94
Age of child	12–23 months	2004	24.6
	> 23 months	6142	75.4
Plurality	Single	7955	97.66
	Multiple	191	2.34
Birth order	≤ 3	3919	48.1
	> 3	4228	51.9
Community level variables			
Distance from health facilities	Not big problem	3199	39.27
	Big problem	4947	60.73
Community education	Low	4230	51.93
	High	3916	48.07
Community media usage	Low	3639	44.67
	High	4508	55.33
Community poverty	Low	4945	60.71
	High	3201	39.29
Residence	Urban	878	10.77
	Rural	7269	89.23
Region	Metropolis	233	2.85
	Large central	7349	90.22
	Small periphery	564	6.93

### Deworming among preschool age children in Ethiopia

**Fig. 1:**
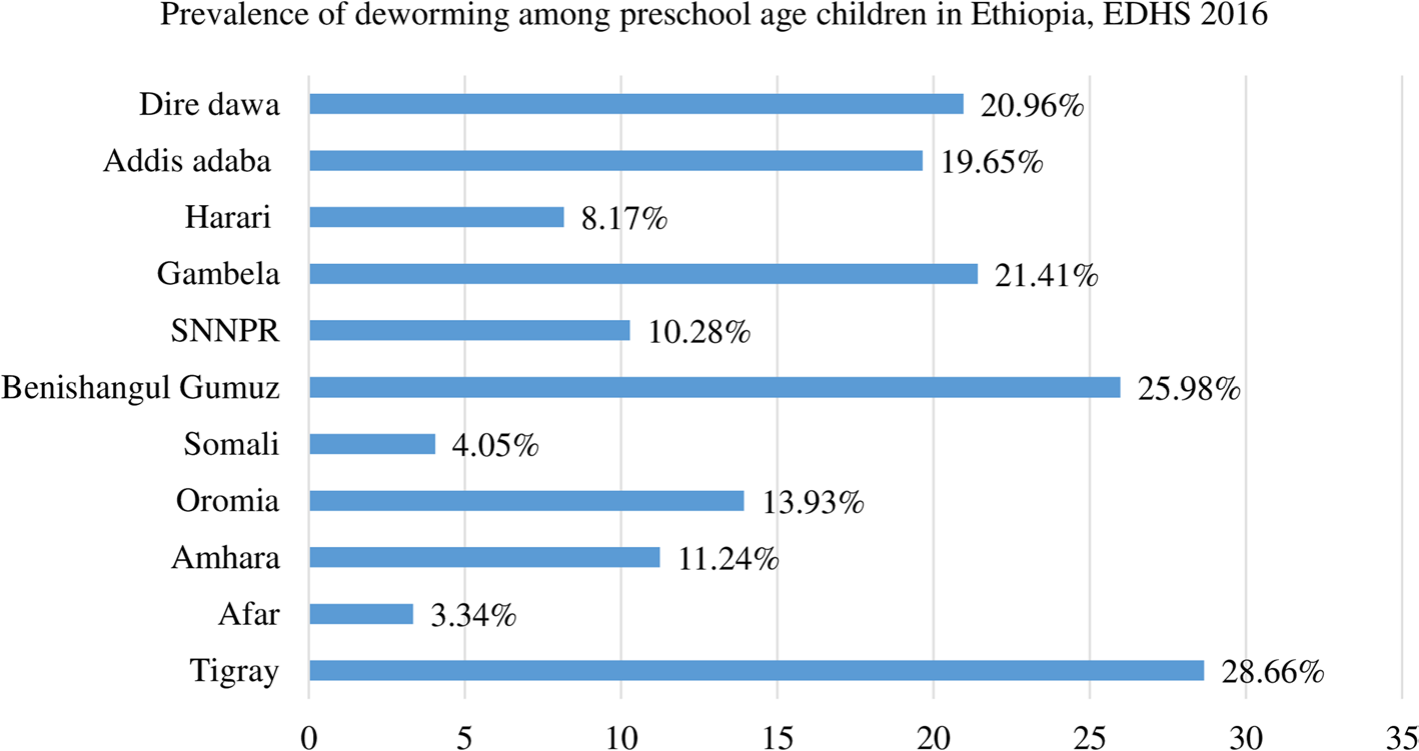
Regional prevalence of deworming among preschool age children in Ethiopia, EDHS 2016

The prevalence of deworming among preschool age children in Ethiopia was 13.32% (95% CI: 12.60, 14.08). The lowest prevalence was seen in the Afar region 3.34% (95% CI: 1.01, 10.45) whereas the highest prevalence was seen in the Tigray region 28.66% (95% CI:24.95, 32.69) [Fig. 1].

### Multi-level analysis of determinant of deworming among preschool age children in Ethiopia

#### Model comparison and random effect analysis

As shown in Table 2, since it has the highest log likelihood (− 1787) and the lowest deviance (3574) value, model 4 in the multilevel analysis is better than all the other multilevel models as well the standard logistics regression model (the model that included all the variables but without random effect).

**Table 2:** Model compression and random effect analysis of deworming among preschool children

Parameters	Standard logistics regression model	Multilevel logistics regression model			
		Null model	Model 2	Model 3	Model 4
Model comparisons					
Log-likelihood	− 1876	− 2943	− 1796	− 2904	**−1787**
Deviance	3752	5886	3592	5808	**3574**
Random effects					
Variance	–	1.45	1.16	1.17	1.15
ICC	–	0.31	0.27	0.28	0.26
MOR	–	4.22	2.78	2.80	2.76
PCV	–	Reference	0.20	0.19	0.21

*ICC* Inter cluster correlation coefficient, *MOR* Median odds ratio, *PCV* proportional change in variance, *VIF* Variance Inflation Factors

The ICC value in the null model was showed 31% of the variations in deworming among preschool children were attributed to cluster differences. The MOR in the null model, also revealed that the median odds ratio between the higher and lower deworming area among clusters was 4.22. Moreover, about 21% of the variation in deworming among preschool children was explained by both community level and individual level variables [Table 2].

#### Fixed effect analysis

In the final model of multilevel logistics regression analysis variables such as; education status of women, occupation of the women, age of the child, ANC visit, media exposure status of the household, and community level media usage had a significant association with deworming of preschool age children. Women who have primary and above primary educational status were 1.50 and 1.89 times more likely to take their child deworming medication than women with no formal education [AOR = 1.50; 95%CI; 1.21, 1.86] and [AOR = 1.89; 95%CI; 1.32, 2.73] respectively. The odds of having deworming among preschool age children whose mothers had worked were 1.47 times higher as compared to children from no worked mothers [AOR = 1.47; 95%CI; 1.23, 1.76].

Children whose age found 24–59 months, were two times more likely to take deworming medication as compared to a child with 12–23 months of age [AOR = 2.00; 95%CI; 1.62, 2.46]. Women who have Anti Natal Care (ANC) were 1.68 times more likely to take their child deworming medication than women with no ANC medication [AOR = 1.68; 95%CI; 1.35, 2.08].

Children who were live in households that have media exposure and live in high usage of community media were 50 and 89% more likely to take deworming medication as compared to households that have no media exposure and community which use no media [AOR = 1.50; 95%CI; 1.22, 1.85] and [AOR = 1.89; 95%CI; 1.31, 2.74] respectively [Table 3].

**Table 3:** Multilevel analysis of factors associated with deworming among children age 0–23 months in Ethiopia, EDHS 2016

Variables	Categories	^a^Model 2	Model 3	Model 4
		AOR [95% CI]	AOR [95% CI]	AOR [95% CI]
Age of women (years)	15–19	1.00	**–**	1.00
	20–35	0.99 [0.76, 1.27]	**–**	0.98 [0.76, 1.28]
	36–49	1.21 [0.87, 1.69]	**–**	1.24 [0.86, 1.68]
Sex of household head	Male	1.00	**–**	1.00
	Female	0.71 [0.53, 0.95]*	**–**	0.75 [0.54, 1.01]
`Educational attainment of women	No education	1.00	**–**	1.00
	Primary education	1.49 [1.21, 1.86]***	**–**	1.50 [1.21, 1.86]*
	Secondary&above	1.87 [1.32, 2.66]***	**–**	1.89 [1.32, 2.73]*
Occupation of women	Not worked	1.00	**–**	1.00
	Worked	1.49 [1.24, 1.78]***	**–**	1.47 [1.23, 1.76]**
Marital status of a mother	Married	1.00	**–**	1.00
	Not married	0.68 [0.46, 1.01]	**–**	0.69 [0.47, 1.02]
Household family size	1–4	1.00	**–**	1.00
	5–10	1.19 [0.95, 1.51]	**–**	1.22 [0.96, 1.53]
	> 11	0.74 [0.39, 1.38]	**–**	0.75 [0.41, 1.42]
Media exposure	No	1.00	**–**	1.00
	Yes	1.51 [1.22, 1.85]***	**–**	1.50 [1.22, 1.85]*
Wealth index	Poorest	1.00	**–**	1.00
	Middle	[0.93, 0.72, 1.19]	**–**	0.92 [0.71, 1.17]
	Richest	1.12 [0.87, 1.44]	**–**	1.03 [0.86, 1.42]
Sex of child	Male	1.00	**–**	1.00
	Female	0.72 [0.54, 0.96]*	**–**	0.81 [0.66, 1.01]
Age of child	12–23 months	1.00	**–**	1.00
	> 24 months	3.02 [2.28, 3.99]***	**–**	2.00 [1.62, 2.46]***
Plurality of birth	Single	1.00	**–**	1.00
	Multiple	0.55 [0.22, 1.35]	**–**	0.52 [0.21, 1.29
Birth Order	< 3	1.00		1.00
	> 3	0.91 [0.71, 1.18]		0.91 [0.70, 1.17]
Pregnancy wontedness	Wanted	1.00	**–**	1.00
	Unwanted	1.07 [0.87, 1.29]	**–**	1.07 [0.87, 1.30]
ANC visits	No ANC	1.00	**–**	1.00
	At least one ANC	1.68 [1.36, 2.08]***	**–**	1.68 [1.35, 2.08]**
Place of delivery	Home delivery	1.00	**–**	1.00
	Health facilities	1.03 [0.82, 1.28]	**–**	1.03 [0.82, 1.30]
Community level variables				
Distance from health facilities	Not big problem	**–**	1.00	1.00
	Big problem	**–**	0.79 [0.67, 0.93]*	0.85 [0.69, 1.04]
Community education	Low	**–**	1.00	1.00
	High	**–**	1.75[1.27, 2.41]**	1.27 [0.89, 1.81]
Community media usage	Low	**–**	1.00	1.00
	High	**–**	2.16 [1.55, 3.02]**	1.89 [1.31, 2.74]*
Community poverty	Low	**–**	1.00	1.00
	High	**–**	0.95 [0.67, 1.33]	0.91[0.62, 1.33]
Residence	Urban	**–**	1.00	1.00
	Rural	**–**	0.57 [0.39, 0.85]*	1.03 [0.64, 1.64]
Region	Metropolis	**–**	1.00	1.00
	Large central	**–**	1.23 [0.71, 2.11]	1.32 [0.73, 2.37]
	Small periphery	**–**	0.65 [0.35, 1.21]	1.05 [0.51, 2.18]

*=P value < 0.05, **=P value < 0.01, ***=P value < 0.001

*AOR* Adjusted Odds Ratio, *CI* Confidence Interval

^*m*^*odel 1(null model)=the model which contains only* with dependent variable and values expressed

### Spatial analysis of deworming among preschool age children in Ethiopia based on 2016 EDHS

**Fig. 2:**
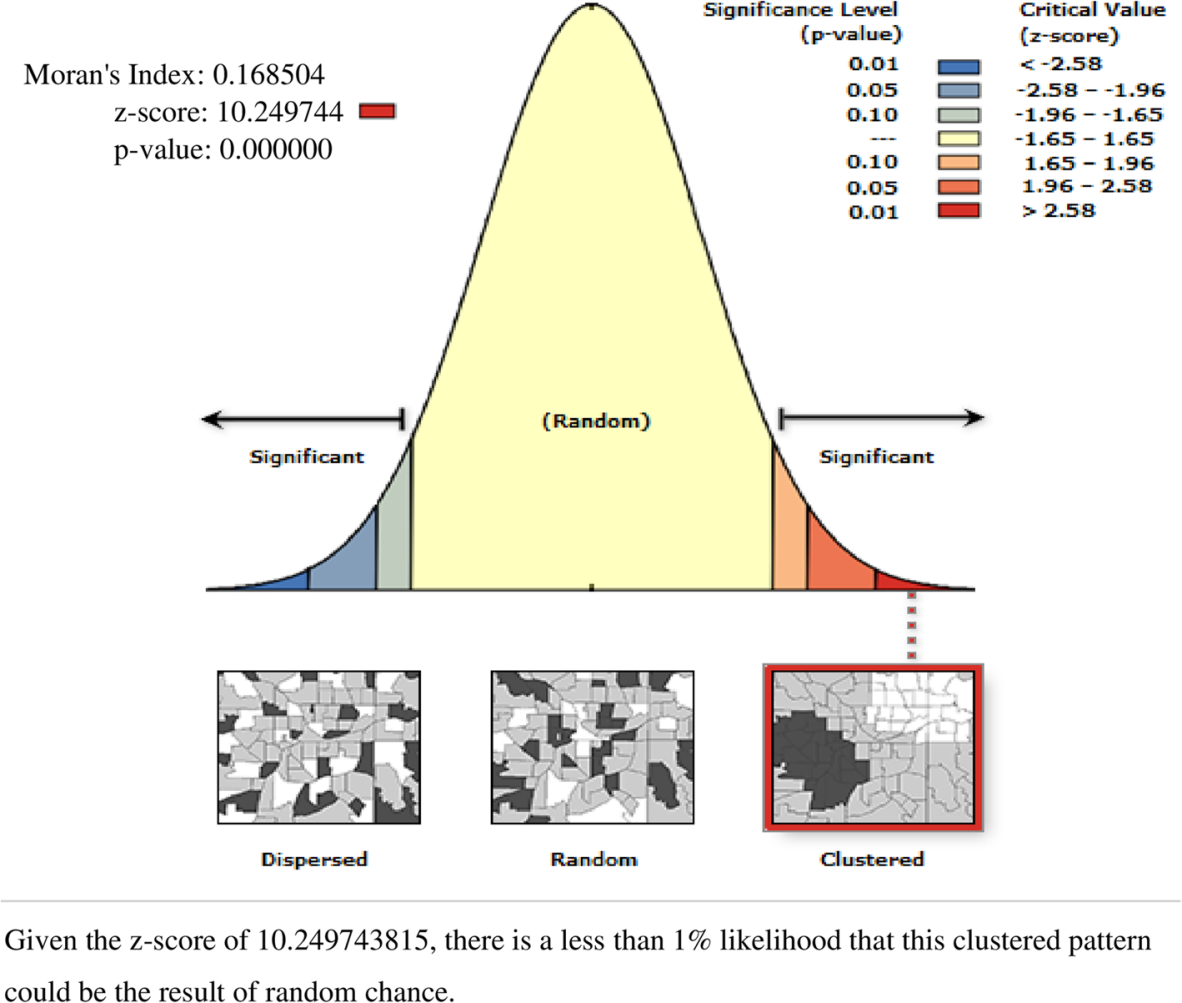
Spatial autocorrelation analysis of deworming among preschool age children in Ethiopia, 2016 EDHS

**Fig. 3:**
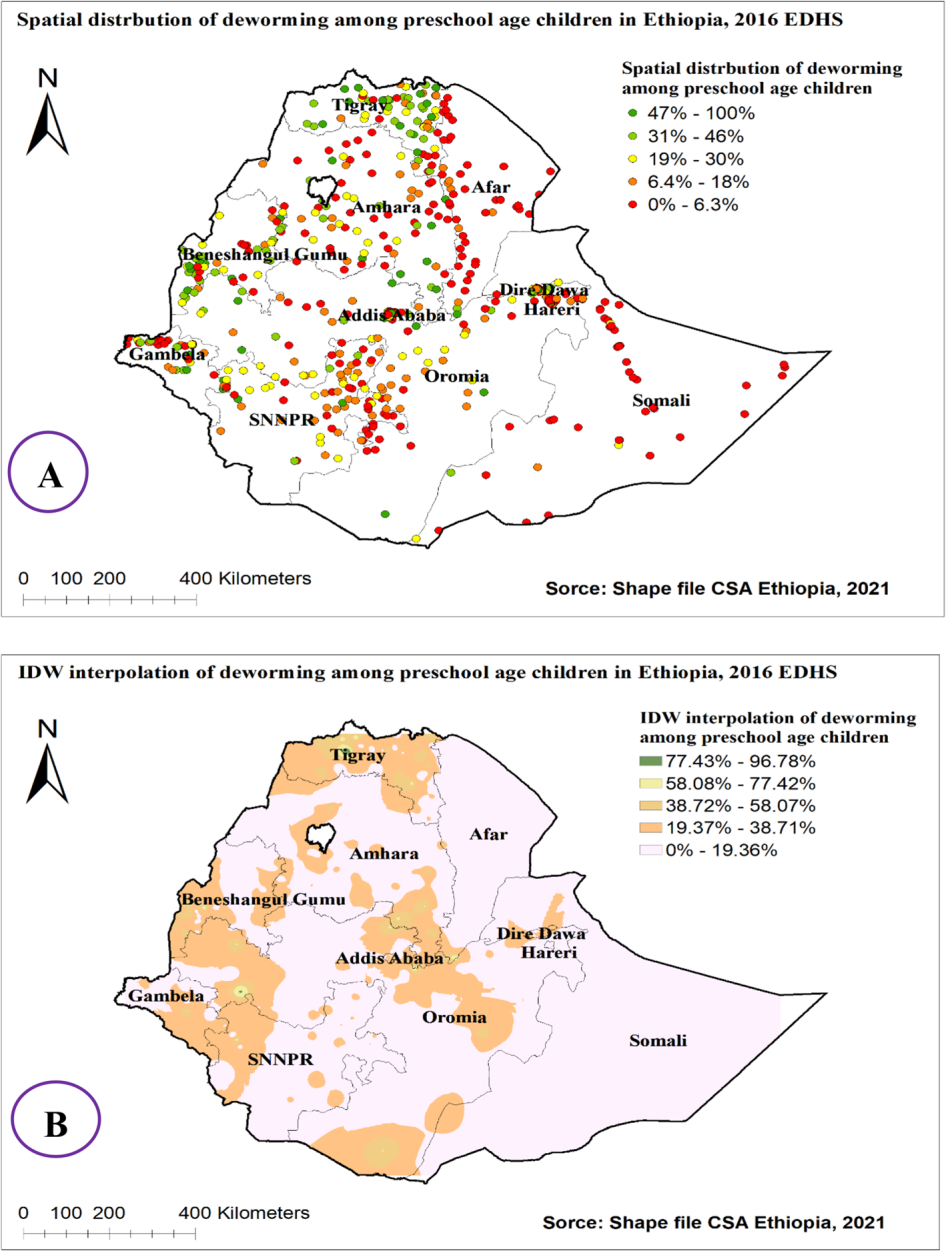
Spatial distribution (**A**) and IDW interpolation (**B**) of deworming among preschool age children in Ethiopia, 2016 EDHS

The spatial distribution of utilization of deworming medication among preschool age children in Ethiopia showed significant clustering over regions in the country, with Global Moran’s I value 0.268 with (*p* < 0.0001). It is more common in Addis Ababa, Tigray, and B/Gumuz [Fig. 2 & 3 (A)]. The incremental autocorrelation result showed that statistically significant z-scores indicated at one peak distance at 196.39 KM; 13.91 (distances; Z-score) for deworming, in which spatial processes promoting clustering are most pronounced detected by 10 distance bands.

The Inverse Distance Weight (IDW) interpolation methods of predicting taking deworming medication among preschool age children in Ethiopia over the area was decreased from green-colored which indicates high utilization of deworming medication to white-colored which shows low utilized areas. The prevalence of low utilization areas deworming medication among preschool age children ranges from 0 to 19.36% and is located in Somalia, Afar, Dire dawa, Harari, Amhara, Oromia, and SNNPE (south nation nationalities and peoples of Ethiopia) regions [Fig. 3 (B)].

### Hot spot area and spatial window analysis of deworming practice among preschool age children in Ethiopia

**Fig. 4:**
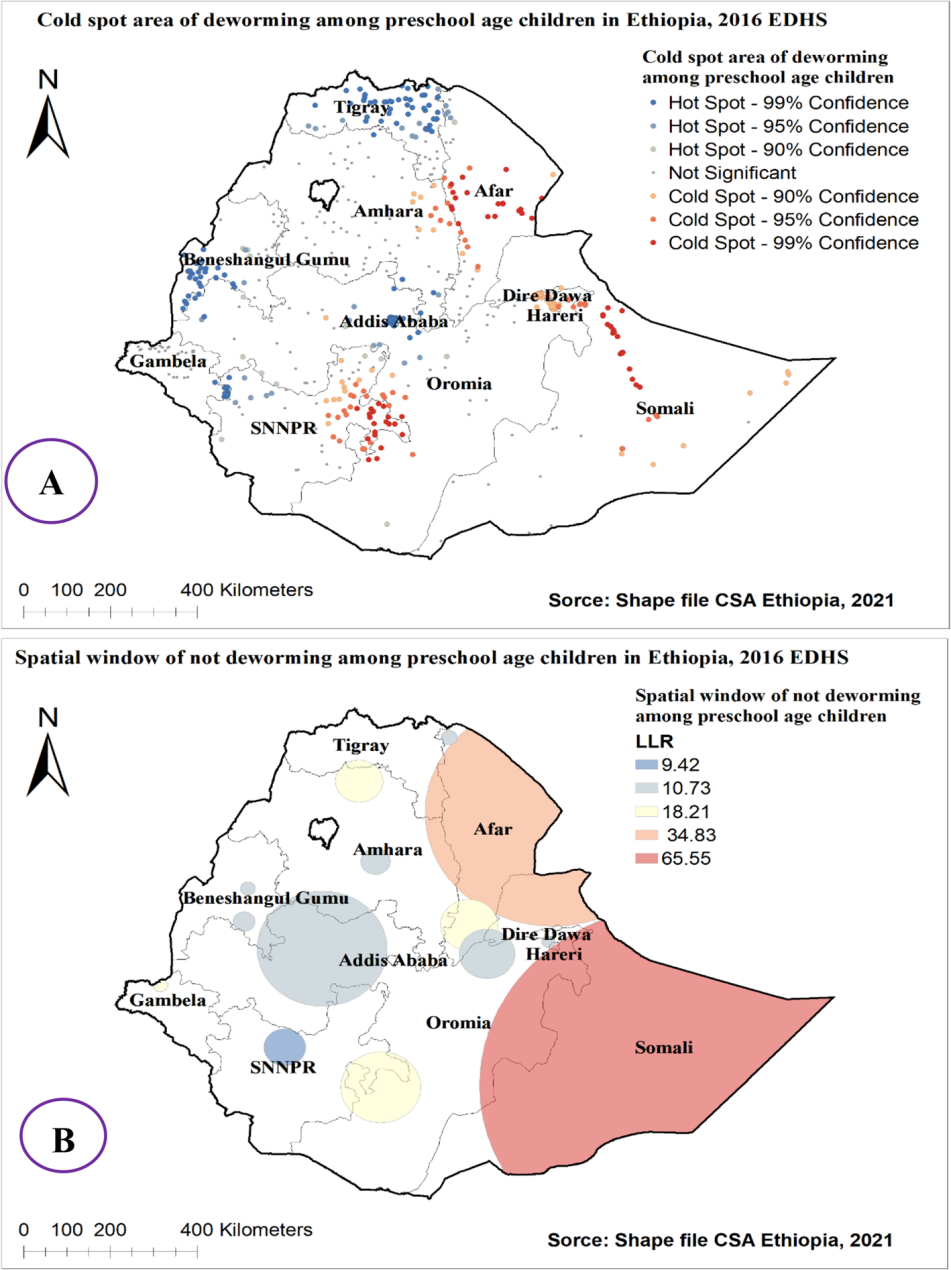
Hot and cold spot area (**A**), and **S**at Scan analysis (**B**) of deworming among preschool age children in Ethiopia, 2016 EDHS

The hot spot analysis of deworming practice among preschool age children in Ethiopia showed that Afar, Eastern Amhara, Diredawa, Harari, Somalia, and Eastern SNNPE regions were cold spot areas of deworming utilization [Fig. 4 (A)].

The SaTScan spatial window analysis showed that 55 primary clusters did not utilize the deworming medication for preschool aged children in Ethiopia. These were located in entire Somalia, Eastern part of Oromia regions, centered at 6.023458 N, 44.807507 E with 466.62 km radius **[**Table 4**].** Children which were found in the primary SaTScan window were 1.17 times more likely to not use deworming medication than out of window regions (RR = 1.17, *P*-value< 0.0001) [Fig. 4 (B)].

**Table 4:** Significant spatial clusters of not taking deworming medication among children age 0–23 months in Ethiopia, EDHS 2016

Clusters [number]	Enumeration areas (clusters) detected	Coordinate/radius	Population	Cases	RR	LLR	P-value
1^ry^[55]	146, 138, 92, 490, 543, 492, 85, 358, 164, 77, 171, 198, 629, 95, 497, 278, 521, 588, 458, 553, 269, 318, 378, 187, 630, 214, 251, 573, 556, 239, 116, 22, 520, 33, 568, 277, 480, 527, 208, 64, 439, 57, 8, 210, 186, 394, 454, 436, 566, 212, 501, 513, 68, 622, 1	6.023458 N, 44.807507 E / 466.62 km	733	711	1.17	65.55	< 0.001
2nd [24]	366, 4, 427, 632, 440, 75, 596, 178, 499, 205, 334, 570, 599, 348, 544, 389, 241, 344, 332, 172, 571, 488, 191, 130, 249, 368, 189, 511, 55, 585, 547, 128, 254, 276, 442, 79, 455, 235, 351, 421, 200, 97, 496, 611, 449, 362, 620, 127	12.401068 N, 42.163134 E / 293.75 km	488	469	1.15	34.83	< 0.001
3rd [18]	316, 232, 398, 600, 182, 32, 574, 21, 445, 34, 468, 634, 313, 405, 422, 576, 215, 408	6.005498 N, 38.525295 E / 92.08 km	211	205	1.15	18.21	< 0.001
4th [10]	39, 336, 564, 484, 102, 135, 37, 295, 51, 283	9.798697 N, 40.380059 E / 66.01 km	118	115	1.15	10.73	0.012
5th [7]	69, 426, 104, 260, 233, 603, 346	8.364914 N, 33.909437 E / 17.31 km	79	78	1.17	9.42	0.036

## Discussions

This study aimed to assess the prevalence and spatial distribution and to identify the community and individual-level factors associated with utilization of deworming among pre SAC in Ethiopia. Based on this, the prevalence of deworming among preschool age children in Ethiopia was 13.32% (95% CI: 12.60, 14.08). This is lower than a study conducted in Kenya (19.6%) [35], in Zambia (93.4%) [36], and Nigeria (42%) [1]. Moreover, our study is lower than a study conducted among pre-school age children from 45 countries in Africa, the Americas, Asia, and Europe (43%), ranging from 6.1% (Azerbaijan) to 87.4% (Rwanda) [37]. This might be due to variations in socio-cultural aspects among study participants and due to differences in awareness levels about the importance of deworming supplementation [16]. Furthermore, mothers’ general familiarity with deworming medication for STH infections in preschool children is a determinant for these differences [1].

In this study, women who attended primary and above primary educational status were more likely to take their child deworming medication as compared to uneducated mothers. This is in line with studies in Cameron [18], and Ghana [19] which show educated mothers were more utilizing deworming medication for their child and themselves. This is because of that, the educated mother has health advantages and better essence of health inputs such as dewormed to the health of children relative to the uneducated partners [19, 38].

In this study, mothers who had worked were more likely to have deworming children. This is in line with a study conducted in Ghana [19], which showed that mothers who have employment were more likely to deworm their children relative to their unemployed counterparts. This might be due to the that, the employed people might have exposure to the importance of supplements and on the other side, the employment rate might depend on education status [16, 19].

In this study, 24–59 months old children were two times more likely to take deworming medication as compared to 12–23 months old children. It is indirectly related to a study in Ethiopia that showed that as the age of a child increases the probability of being anemic becomes decreases [20]. The utilization of deworming directly prevents anemia among pre SAC by preventing hookworm infection [39]. This might be because most mothers think that, the child can resist medication side effect when the age becomes increase. On the other hand, most people perceived that the best time to deworm a child and give other supplements is right from the age of two [40].

Women who have Anti Natal Care (ANC) visits were more likely to take their child deworming medication than women with no ANC visit. A study in 26 sub-Saharan African countries for utilization of deworming for pregnant mothers showed that having ANC visits has a positive association with utilization of deworming medication [41]. So as if women utilized a deworming medication for themselves because of havening ANC follow up, they already know the advantage and expected to use it for their child.

Children who were lived in households that have media exposure and live in a community that has high media usage were 50 and 89% more likely to take deworming medication as compared to their counterparts respectively. It is in line with a study in India [17] and supported by a study done in Nigeria which showed that utilization of health care can be improved when maternal media exposure increases [1]. Moreover, a study in 26 sub-Saharan African countries showed that women who have media exposure had higher odds of the utilization of deworming for themselves [41]. Exposure to media could have a tremendous role in increasing awareness and knowledge for mothers and the dissemination of health-related information [41].

The spatial analysis results in this study showed that utilization of deworming medication among pre SAC children in Ethiopia was not randomly distributed over regions in the country. It is more common in Addis Ababa, Tigray, and B/Gumuz regions. But in Somalia, Afar, Amhara, Oromia, and SNNPE were the lowest utilization area of deworming medication among pre SAC children. This is supported by a study in Kenya that showed that STH infections showed micro-geographical heterogeneities [42]. Therefore the utilization and regularity of deworming might depend on the spatial distribution of the disease.

The main strength of this study was the use of the weighted nationally representative data with a large sample which makes it representative at country levels. Therefore, it has appropriate statistical power that can be generalized of the estimates in deworming pre SAC during the study period. Since the data were collected cross-sectional by self-reported interview would be prone to recall and social desirability bias. The drawback of the secondary nature of data was inevitable.

## Conclusions

The utilization of deworming medication among preschool age children in Ethiopia is relatively low. Individual level factors such as; maternal education and occupation, having ANC visit, child age, household media exposure, and community level variables such as having high community media usage were significant predictors of deworming supplementation. Afar, Eastern Amhara, Dire Dewa, Somalia, and Eastern SNNPE regions were spatial cold spot areas for deworming among preschool age children in Ethiopia. These findings highlight that, the Ministry of Health (MOH) Ethiopia should promote deworming in mass media and prepare regular programs for deworming of children in campaigns. Ministry of Education should work as an integrated approach with other stalk holders to strengthen women’s education, household and community media exposure. Prior attention should be given to those areas which have low utilization of deworming medication such as Somalia, Diredewa, Afar, Amhara, and SNNPE regions.

There are no financial, non-financial, and commercial organizations competing of interests.

## Data Availability

Data is available publically access from the open databases. It can be accessed by the following website.https://dhsprogram.com/data/dataset_admin/login_main.cfm?CFID=10818526&CFTOKEN=c131014a480fe56-4E0C6B7F-F551-E6B2-50

## Abbreviations

AOR: Adjusted Odds Ratio
ANC: Anti Natal Care
CI: Confidence Interval
CSA: Central Statistical Agency
EDHS: Ethiopian Demographic and Health Survey
KR: Kids Record
MOH: Ministry Of Health
pre SAC: Pre School Age Children
SDG: Sustainable Development Goal
STH: Soil-Transmitted Helminthic
WHO: World Health Organization

## References

[1] Eze P (2020). Perception and attitudinal factors contributing to periodic deworming of preschool children in an urban slum, Nigeria. BMC Public Health.

[2] Becker SL (2018). Toward the 2020 goal of soil-transmitted helminthiasis control and elimination. PLoS Negl Trop Dis.

[3] Taylor-Robinson DC (2019). Public health deworming programs for soil-transmitted helminths in children living in endemic areas. Cochrane Database Syst Rev.

[4] Tchuenté LT (2011). Control of soil-transmitted helminths in sub-Saharan Africa: diagnosis, drug efficacy concerns, and challenges. Acta Trop.

[5] Kumapley RS, Kupka R, Dalmiya N (2015). The role of child health days in the attainment of global deworming coverage targets among preschool-age children. PLoS Negl Trop Dis.

[6] Ali J (2019). Deworming school children in Ethiopia: the importance of a comprehensive approach. Open J Trop Med.

[7] Chelkeba L (2020). Epidemiology of intestinal parasitic infections in preschool and school-aged Ethiopian children: a systematic review and meta-analysis. BMC Public Health.

[8] Nute AW (2018). Prevalence of soil-transmitted helminths and Schistosoma mansoni among a population-based sample of school-age children in Amhara region, Ethiopia. Parasites Vectors.

[9] Crompton DWT, Nesheim MC (2002). Nutritional impact of intestinal helminthiasis during the human life cycle. Annu Rev Nutr.

[10] Gadisa E, Jote K (2019). Prevalence and factors associated with intestinal parasitic infection among under-five children in and around Haro Dumal town, bale zone, Ethiopia. BMC Pediatr.

[11] Belachew A, Tewabe T (2020). Under-five anemia and its associated factors with dietary diversity, food security, stunted and deworming in Ethiopia: systematic review and meta-analysis. Syst Rev.

[12] Awasthi S (2008). Effects of deworming on malnourished preschool children in India: an open-labeled, cluster-randomized trial. PLoS Negl Trop Dis.

[13] Helminthiases WS-T (2012). Eliminating soil-transmitted helminthiases as a public health problem in children: progress report 2001–2010 and strategic plan 2011–2020. France: world health. Organization.

[14] World Health Organization. Guideline: preventive chemotherapy to control soil-transmitted helminth infections in at-risk population groups. 2017. https://www.who.int/elena/titles/full_recommendations/deworming/en/. 29578660

[15] Lo NC, Heft-Neal S, Coulibaly JT, Leonard L, Bendavid E, Addiss DG. Global state of deworming coverage and inequity in low-income and middle-income countries: a spatiotemporal study of household health surveys. bioRxiv. 2019;589127. 10.1016/S2214-109X(19)30413-9 PMC7024997 31558383

[16] Mulaw GF (2021). Deworming coverage and its predictors among Ethiopian children aged 24 to 59 months: further analysis of EDHS 2016 data set. Global. Pediatr Health.

[17] Ali B, Chauhan S (2020). Inequalities in the utilization of maternal health care in rural India: pieces of evidence from national family health survey III & IV. BMC Public Health.

[18] Zegeye B (2021). Utilization of deworming drugs and its individual and community level predictors among pregnant married women in Cameroon: a multilevel modeling. Biomed Res Int.

[19] Immurana M, Arabi U (2016). Socio-economic covariates of micronutrients supplementation and deworming among children in Ghana. J Behav Health.

[20] Gebremeskel MG (2020). Individual and community level factors associated with anemia among children 6—59 months of age in Ethiopia: a further analysis of 2016 Ethiopia demographic and health survey. PLoS One.

[21] Rosen G (2015). A history of public health.

[22] Heck RH, Thomas SL, Tabata LN (2013). Multilevel and longitudinal modeling with IBM SPSS.

[23] African Countries by Population. Worldometer; 2021. https://www.worldometers.info/population/countries-in-africa.

[24] Demographic and Health Survey (DHS). USAID from the American people, ICF, 530 Gaither Road, Suite 500, Rockville, MD 20850: https://dhsprogram.com/Methodology/Survey-Types/DHS.cfm.

[25] Croft (2018). Guide to DHS Statistics.

[26] Central Statistical Agency Addis Ababa, E (2017). ETHIOPIA Demographic and Health Survey 2016.

[27] Teshale AB, Tesema GA (2020). Magnitude and associated factors of unintended pregnancy in Ethiopia: a multilevel analysis using 2016 EDHS data. BMC Pregnancy Childbirth.

[28] Teshale AB, Tesema GA (2020). Prevalence and associated factors of delayed first antenatal care booking among reproductive-age women in Ethiopia; a multilevel analysis of EDHS 2016 data. PLoS One.

[29] Liyew AM, Teshale AB (2020). Individual and community level factors associated with anemia among lactating mothers in Ethiopia using data from Ethiopian demographic and health survey, 2016; a multilevel analysis. BMC Public Health.

[30] Midi H, Sarkar SK, Rana S (2010). Collinearity diagnostics of the binary logistic regression model. J Interdiscip Math.

[31] Merlo J (2005). A brief conceptual tutorial of multilevel analysis in social epidemiology: linking the statistical concept of clustering to the idea of contextual phenomenon. J Epidemiol Community Health.

[32] Merlo J (2005). A brief conceptual tutorial on multilevel analysis in social epidemiology: interpreting neighborhood differences and the effect of neighborhood characteristics on individual health. J Epidemiol Community Health.

[33] McMillen DP (2004). Geographically weighted regression: the analysis of spatially varying relationships.

[34] Kulldorff M (1997). A spatial scan statistic. Commun Stat Theory Methods.

[35] Clohossey PC (2014). Coverage of vitamin a supplementation and deworming during Malezi bora in Kenya. J Epidemiol Glob Health.

[36] Babaniyi O, Siziya S, Mukonka V, Kalesha P, Mutambo H, Matapo B, Musanje H. Child nutrition and health campaign in 2012 in Zambia: coverage rates for measles, Oral polio vaccine, vitamin a, and De-worming. The Open Vaccine Journal. 2013;6(1-8).

[37] Lo NC (2018). Deworming in pre-school age children: a global empirical analysis of health outcomes. PLoS Negl Trop Dis.

[38] Abeway S (2018). Stunting and its determinants among children aged 6–59 months in northern Ethiopia: a cross-sectional study. J Nutr Metab.

[39] Girum T, Wasie A (2018). The effect of deworming school children on anemia prevalence: a systematic review and meta-analysis. Open Nurs J.

[40] Parenting Desk. Why deworming is necessary for your child. New Delhi: The Indian Express; 2022. https://indianexpress.com/article/parenting/health-fitness/why-deworming-is-necessary-for-your-child-7186018/.

[41] Zegeye B (2021). Utilization of deworming medication and its associated factors among pregnant married women in 26 sub-Saharan African countries: a multi-country analysis. Trop Med Health.

[42] Chadeka EA (2017). Spatial distribution and risk factors of Schistosoma haematobium and hookworm infections among schoolchildren in Kwale, Kenya. PLoS Negl Trop Dis.

